# Association of surrogate adiposity markers with prevalence, all-cause mortality and long-term survival of heart failure: a retrospective study from NHANES database

**DOI:** 10.3389/fendo.2025.1430277

**Published:** 2025-03-04

**Authors:** Fan-Shun Guo, Chen Guo, Jia-Hao Dou, Jun-Xiang Wang, Rui-Yun Wu, Shou-Fang Song, Xue-Lu Sun, Yi-Wei Hu, Jin Wei

**Affiliations:** ^1^ Department of Cardiology, The Second Affiliated Hospital of Xi’an Jiaotong University, Xi’an, Shaanxi, China; ^2^ Clinical Research Center for Endemic Disease of Shaanxi Province, The Second Affiliated Hospital of Xi’an Jiaotong University, Xi’an, Shaanxi, China; ^3^ Medicine Department of Xi’an Jiaotong University, Xi’an, Shaanxi, China

**Keywords:** surrogate adiposity markers, heart failure, abdominal obesity, NHANES, retrospective study

## Abstract

**Introduction:**

Obesity, especially abdominal obesity, is more common in patients with heart failure (HF), but body mass index (BMI) cannot accurately describe fat distribution. Several surrogate adiposity markers are available to reflect fat distribution and quantity. The objective of this study was to explore which adiposity marker is most highly correlated with HF prevalence, all-cause mortality and patients’ long-term survival.

**Methods:**

The National Health and Nutrition Examination Survey (NHANES) database provided all the data for this study. Logistic regression analyses were adopted to compare the association of each surrogate adiposity marker with the prevalence of HF. Cox proportional hazards models and restricted cubic spline (RCS) analysis were employed to assess the association between surrogate adiposity markers and all-cause mortality in HF patients. The ability of surrogate adiposity markers to predict long-term survival in HF patients was assessed using time-dependent receiver operating characteristic (ROC) curves.

**Results:**

46,257 participants (1,366 HF patients) were encompassed in this retrospective study. An area under the receiver operating characteristic curve (AUC) for the prevalence of HF assessed by weight-adjusted-waist index (WWI) was 0.70 (95% CI: 0.69-0.72). During a median follow-up of 70 months, 700 of 1366 HF patients’ death were recorded. The hazard ratio (HR) for HF patients’ all-cause mortality was 1.33 (95% CI: 1.06-1.66) in the a body shape index (ABSI) quartile 4 group and 1.43 (95% CI: 1.13-1.82) in the WWI quartile 4 group, compared with the lowest quartile group. The AUC for predicting 5-year survival of HF patients using the ABSI was 0.647 (95% CI: 0.61-0.68).

**Conclusions:**

WWI is strongly correlated with the prevalence of HF. In HF patients, those with higher WWI and ABSI tend to higher all-cause mortality. ABSI can predict patients’ long-term survival. We recommend the use of WWI and ABSI for assessing obesity in HF patients.

## 1 Introduction

Heart failure (HF) is considered the terminal stage of cardiovascular diseases (1), with its prevalence and disease burden increasing annually, affecting approximately 64 million individuals worldwide (2–4). Similar to malignancies, HF patients’ long-term survival is not optimistic (5). Data from Get With The Guidelines-Heart Failure (GWTG-HF) and Medicare shows the median survival for inpatients with HF is 2.1 years (6). In spite of the advent of new pharmaceuticals for HF treatment, the 5-year survival rate of inpatients is only 25% (4). Consequently, early diagnosis and long-term prognosis prediction are more crucial for HF patients.

Obesity, notably abdominal obesity, is globally recognized as a significant health concern and the predominant independent risk factors for the development and progression of HF (7–9). Obesity is strongly linked to HF and HF-related disorders, such as hypertension and diabetes mellitus (DM) (10, 11). Although body mass index (BMI) is a common anthropometric index used to describe obesity, it fails to accurately describe fat distribution (12, 13). Paradoxically, a higher BMI is related to a longer survival time when used to predict HF patients prognosis, and this manifestation has been referred to “obesity paradox” (14). Although waist circumference (WC) as well as waist-to-height ratio (WHtR) are currently crucial markers for abdominal obesity (15), they do not offer significant advantages over BMI in predicting the risk and prognosis of cardiovascular diseases (16).

To enhance accuracy in describing body size and fat distribution, several surrogate adiposity markers have been proposed (17–21). The weight-adjusted waist index (WWI) offers a comprehensive evaluation of adiposity, muscle mass, and bone mass (22). Recent research indicates a potential link between higher WWI and increased risk of cardiovascular events in both American and Asian populations (18, 23). A Body Shape Index (ABSI), another index according to waist circumference, height and weight, provides a better evaluation of fat distribution and is positively correlated with adult mortality (17, 24). Visceral adiposity index (VAI) and lipid accumulation product (LAP) describe lipid accumulation and fat distribution, are strongly linked to cardiovascular metabolism and are more effective than BMI in identifying cardiovascular disease risk (20, 21). Relative fat mass (RFM) is also an emergent index with high predictability for metabolic syndrome (MetS) (19). However, limited studies exist comparing these markers in terms of HF prevalence and prognosis.

The aims of this study were to explore which adiposity marker is most strongly associated with HF prevalence and patients’ all-cause mortality and long-term survival. The findings could lead to the adoption of surrogate adiposity markers for obesity assessment and survival prediction in HF patients.

## 2 Materials and methods

### 2.1 Study participants

Data from 1999-2018 of the National Health and Nutrition Examination Survey (NHANES), which was approved by the National Center for Health Statistics (NCHS), were used in this study. All participants signed written informed consent. Within adults aged 20 years and above, we excluded those who (1) had missing information on WC, weight, or BMI (2); did not provide a self-reported history of HF (3); lacked linked mortality data (4); had missing laboratory data. This study ultimately included 46,257 participants, of whom 1,366 had HF (**Figure 1** in **Supplementary Data Sheet 1**). This study constituted a quadratic analysis of publicly available NHANES data, thus not requiring ethical review.

### 2.2 Definition of surrogate adiposity markers, outcomes

The study variables included eight surrogate adiposity markers: BMI, WC, WHtR, WWI, ABSI, LAP, VAI, and RFM. During the survey, professional investigators measured the height, weight, and WC of participants. The formulas for other surrogate adiposity markers are reported in **Table 1** in **Supplementary Data Sheet 1** (17–21).

The main outcomes were whether the participants had a diagnosis of HF and HF patients’ all-cause mortality. Participants’ self-reported history of HF was used to define HF patients. We linked the data from this study to the National Death Index to obtain survival information for participants. Survival time is from when the participant took the survey until either the end of follow-up (December 31, 2019) or death.

### 2.3 Covariates

NHANES categorized race and ethnicity of participants as Mexican American, Other Hispanic, Non-Hispanic White, Non-Hispanic Black, or Other Race based on individual choice. Demographics covariates included sex (female and male), age, marital status (married, widowed, divorced, single, or others), and educational attainment (below high school, high school or general equivalency diploma [GED], above high school, or others). Smoking status was classified into three categories: never smoker (< 100 cigarettes smoked in a lifetime), ever smoker (≥ 100 cigarettes smoked in a lifetime but now quit) and current smoker(≥ 100 cigarettes smoked in a lifetime and not quit). Alcohol consumption was divided into drinking and non-drinking according to the participant’s answer to question, “Do you consume at least 12 drinks per year?”. The ‘others’ category for both demographic covariates and alcohol consumption indicated participants who did not answer the corresponding questions. DM was recognized as the presence of at least one of the following (1): hemoglobin A1c (HbA1c) ≥ 6.5% (2), random blood glucose ≥ 200mg/dL (3), self-reported doctor-diagnosed DM (4), use of insulin or hypoglycemic drugs. Hypertension was characterized by a doctor-reported history of hypertension, systolic blood pressure ≥140 mmHg, or diastolic blood pressure ≥90 mmHg. The laboratory examination data were extracted directly from NHANES.

### 2.4 Statistical analysis

Sampling weights were calculated for this study considering the intricate sampling design of NHANES spanning a 20-year period. Participants were divided into two categories to compare their baseline characteristics based on whether they had HF. Continuous variables were portrayed as weighted means or medians, while categorical variables were depicted as unweighted frequencies (weighted percentages). Continuous variables were analyzed by analysis of variance (ANOVA) or Kruskal-Wallis test and χ^2^ test for categorical variables. Risk factors for HF were assessed using logistic regression analysis.

Participants were grouped according to the quartiles of each surrogate adiposity marker. Three multivariable logistic regression models were developed to assess the correlation between surrogate adiposity markers and HF, utilizing receiver operating characteristic (ROC) curve and an area under ROC curve (AUC) to gauge the predictive accuracy of different surrogate adiposity markers. Moreover, Kaplan-Meier (K-M) method was utilized to illustrate survival trends in HF patients, with the Log-Rank test employed to compare overall patient survival discrepancies across adiposity marker groups. Three multivariable Cox proportional hazard regression models were developed for estimating the connection between surrogate adiposity marker and all-cause mortality in HF patients, presenting outcomes as hazard ratios (HRs) with 95% confidence intervals (CIs). Additionally, restricted cubic spline (RCS) analysis was used to capture the drain-response relations between surrogate adiposity markers and HF patients’ all-cause mortality. Evaluation of the predictive value of surrogate adiposity markers for 1-, 3-, and 5-year survival in HF patients using time-dependent ROC curves. In addition, we performed subgroup analyses to assess the concordance of the prognostic value of BMI, WWI, and ABSI with the primary outcome in HF patients. Subgroup analyses took into account gender, age, race, and the presence of comorbidities. All analyses were performed using R software (version 4.2.2) and SPSS statistical software (version 27.0), with statistical significance set at a two-sided P-value less than 0.05.

## 3 Results

### 3.1 Baseline characteristics and risk factors for HF

The baseline characteristics of the 46,257 (of whom 1,366 participants had HF) participants included in this study are summarized in **Table 2** in **Supplementary Data Sheet 1**. Compared to the Non-HF group, the HF group were older, male, higher percentage widowed, less educated, and fewer never smoked. Interestingly, a lower proportion of participants in the HF group consumed alcohol. Levels of glucose, HbA1c, and triglyceride (TG) were higher in the HF group in contrast to the Non-HF group, while the opposite trend was observed for total cholesterol (TC) and high-density lipoprotein cholesterol (HDL-C). All surrogate adiposity markers differed significantly between the two groups (all P-value < 0.001). HF patients had higher rates of cardiovascular disease (coronary heart disease (CHD), hypertension, myocardial infarction (MI), angina) and DM.

**Figure 1:**
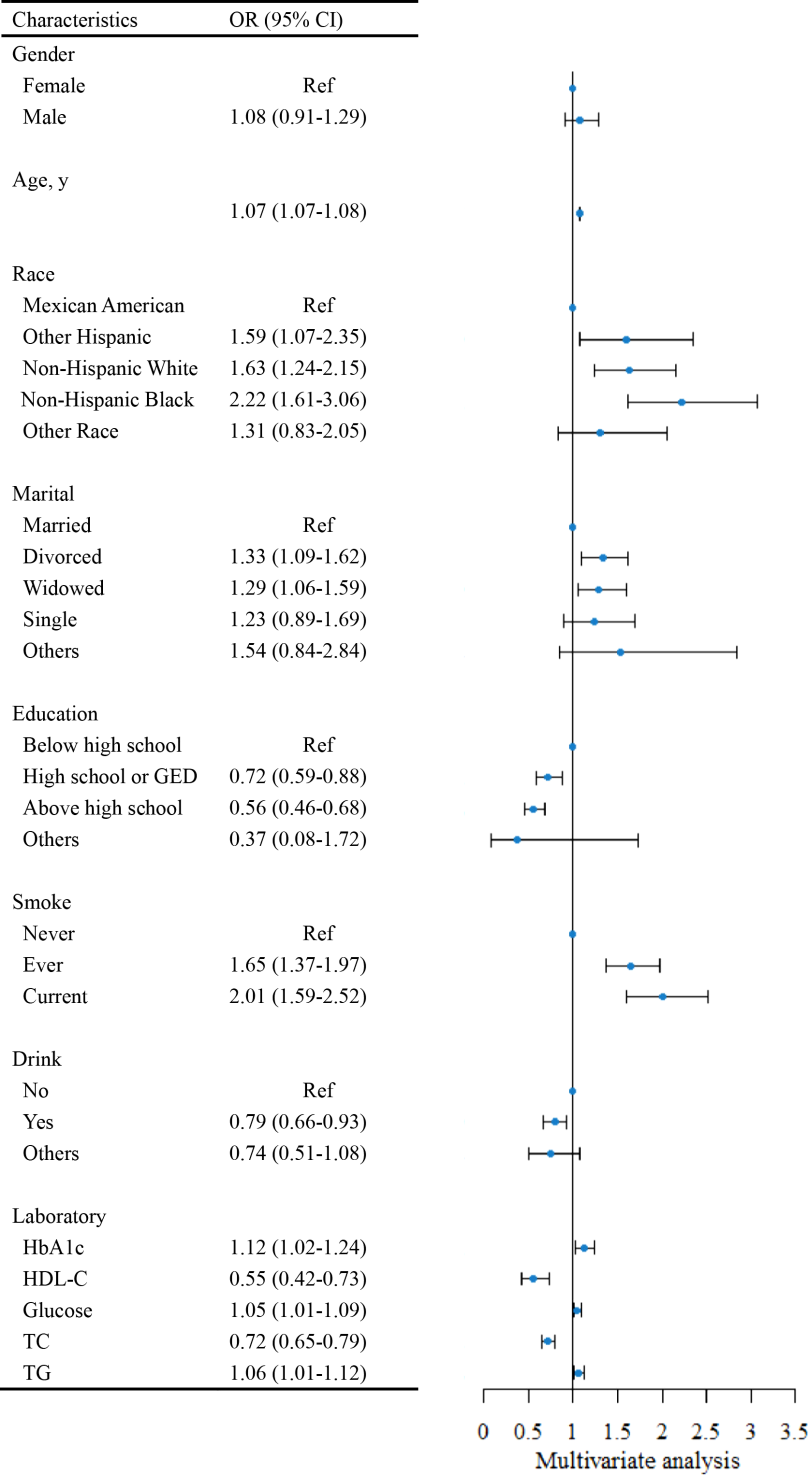
The forest plot for assessing risk factors for Heart Failure (HF). HbA1c, hemoglobin A1c; HDL-C, high-density lipoprotein cholesterol; TC, total cholesterol; TG, triglyceride; Ref, reference; OR, odds ratio. SI conversion factors: To convert HDL-C, glucose, TC, and TG to mmol/L, multiply values by 0.02586, 0.0555, 0.0259, and 0.0113.

Risk factors for HF were investigated using multivariate logistic regression analysis, the results of which are presented in **Figure 1**. Notably, to emphasize the effect of changes in the levels of glucose, TC, HDL-C, and TG on HF, we converted these indices to the International System of Units (SI) in this analysis. Age was a risk factor for HF, with the risk increasing by 7% (95% CI = 1.07-1.08) for each additional year. Compared with Mexican Americans, other Hispanic (OR=1.59, 95% CI = 1.07-2.35), non-Hispanic white (OR=1.63, 95% CI = 1.24-2.15), and non-Hispanic black (OR=2.22, 95% CI = 1.61-3.06) individuals had an elevated risk of HF. Divorced (OR=1.33, 95% CI = 1.09-1.62) and widowed (OR=1.29, 95% CI = 1.06-1.59) participants had an increased risk of HF relative to married respondents. As long as the participant had a history of smoking, the risk of HF increased, regardless of whether they were currently quitting smoking. Higher educational attainment (high school or GED [OR=0.72, 95% CI = 0.59-0.88], above high school [OR=0.56, 95% CI = 0.46-0.68]) was a protective factor for a participant’s risk of HF. Interestingly, alcohol consumption appeared to lower the risk of HF (OR=0.79, 95% CI = 0.66-0.93). Each 1% increase in HbA1c was connected with a 12% (95% CI = 1.02-1.24) higher risk of HF. For every 1 mmol/L increase in glucose and TG, the risk of HF increased by 5% (95% CI = 1.01-1.09) and 6% (95% CI = 1.01-1.12), respectively. An increase in HDL-C and TC was correlated with a decreased risk of HF.

### 3.2 Association of surrogate adiposity markers with HF

**Figure 2:**
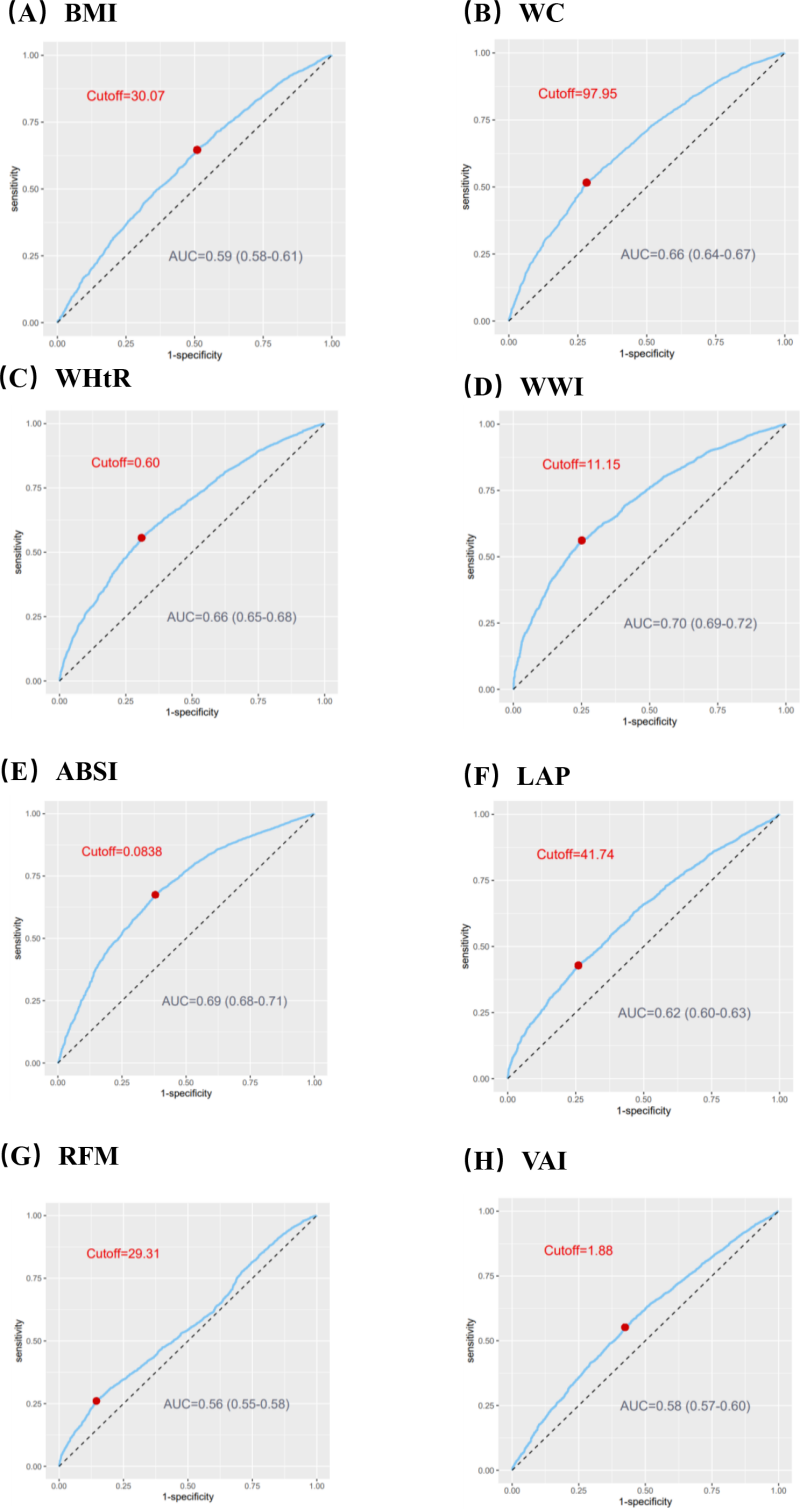
ROC curves to assess the capacity of surrogate adiposity markers to predict the Heart Failure (HF) prevalence. **(A)** Body mass index (BMI). **(B)** Waist circumference (WC). **(C)** Waist-to-height ratio (WHtR). **(D)** Weight-adjusted-waist index (WWI). **(E)** A body shape index (ABSI). **(F)** Lipid accumulation product (LAP). **(G)** Relative fat mass (RFM). **(H)** Visceral fat index (VAI). ROC, receiver operating characteristic; AUC, an area under ROC curve.

Taking the quartiles of surrogate adiposity markers as categorical variables, three models were developed to analyze the relationship between surrogate adiposity markers and HF (**Table 3** in **Supplementary Data Sheet 1**). In model 1, unadjusted for any variables, a rise in each adiposity marker was linked to an enhanced risk of HF development compared to the lowest quadrant. After multivariate adjustment in model 3, all markers, except ABSI and VAI, exhibited an elevated risk of HF prevalence compared to the reference group (P-trend < 0.001). Furthermore, we used ROC curves to assess the risk of HF incidence predicted by different surrogate adiposity markers, the results of which are shown in **Figure 2**. The WWI had the highest AUC of 0.70 (95% CI = 0.69-0.72) and the cutoff point was 11.15.

### 3.3 HF patients’ all-cause mortality with surrogate adiposity markers

During a median follow-up of 70 months, there were 700 deaths among the 1,366 HF patients. All-cause mortality was compared between quartile groups for each surrogate adiposity marker using K-M survival analysis. Patients with higher WWI and ABSI markers had a significantly greater probability of survival than those with lower markers (all Log-Rank P < 0.0001) (**Figures 2B, C** in **Supplementary Data Sheet 1**). In contrast, patients with a lower BMI had a greater probability of survival (Log-Rank P < 0.0001) (**Figure 2A** in **Supplementary Data Sheet 1**). No statistically significant differences were noted for the other surrogate adiposity markers (all Log-Rank P > 0.05) (**Figure 3** in **Supplementary Data Sheet 1**).

**Figure 3:**
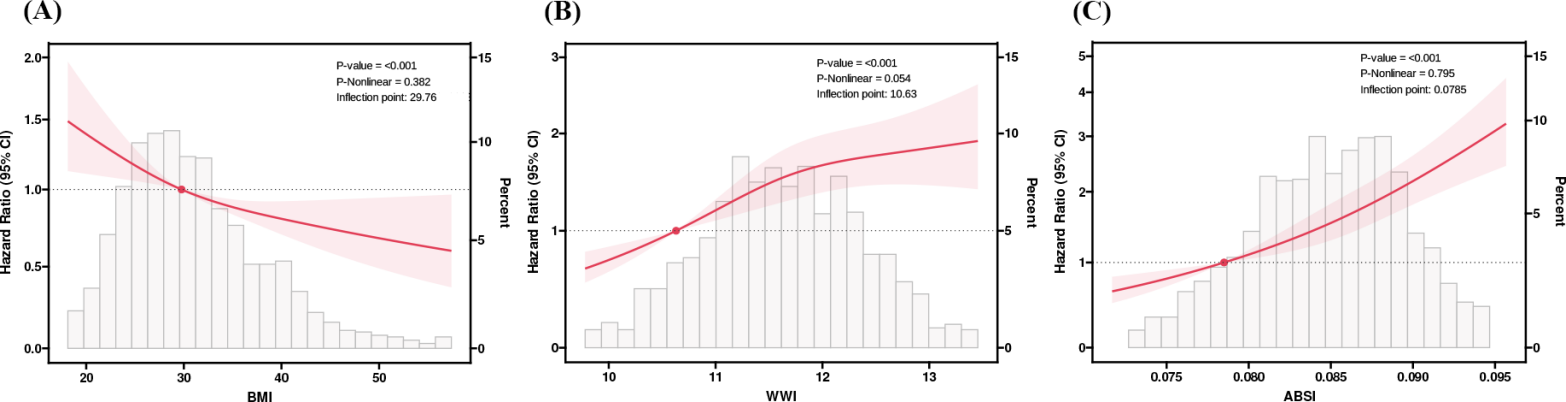
RCS analysis of BMI, WWI, and ABSI with Heart Failure (HF) patients’ all-cause mortality. **(A)** Body mass index (BMI). **(B)** Weight-adjusted-waist index (WWI). **(C)** A body shape index (ABSI). We used RCS with 3 knots at the 10th, 50th and 90th percentiles of BMI, WWI, and ABSI. Hazard ratios (solid lines), 95%Cis (shaded areas). RCS, restricted cubic spline.

Taking the quartiles of BMI, WWI and ABSI as categorical variables, three Cox proportional hazard regression models were employed to evaluate the relationship between these three surrogate adiposity markers and HF patients’ all-cause mortality (**Table 1**). In model 1, higher WWI quartiles, ABSI quartiles and lower BMI quartiles were correlated with higher all-cause mortality rates (P -trend < 0.001). In model 3 with multivariate correction, compared to the reference group, the HR in the fourth quartile of WWI was 1.33 (95% CI = 1.06-1.66, P-trend = 0.04) while in the fourth quartile of ABSI was 1.43 (95% CI = 1.13-1.82, P-trend = 0.003). Conversely, no significant correlation was found between the increase of BMI and changes in all-cause mortality of HF patients after multivariable correction (P for trend = 0.22). The RCS model showed an L-shaped correlation of BMI and ABSI with all-cause mortality in HF patients, while the WWI showed a Log-shaped association (**Figure 3**). The RCS curves were used to identify the inflection points of each of the three markers, and the data were divided into 2 groups for separate regression analyses; the results are shown in **Table 4** in **Supplementary Data Sheet 1**.

**Table 1:** Association of Heart Failure (HF) patients’ all-cause mortality with BMI, WWI, and ABSI.

Characteristics	Model 1^a^ HR (95%CI)	Model 2^b^ HR (95%CI)	Model 3^c^ HR (95%CI)
BMI (quartiles)			
Q1	1 (Ref)	1 (Ref)	1 (Ref)
Q2	0.73 (0.60-0.89) **	0.76 (0.62-0.93) **	0.76 (0.62-0.93) **
Q3	0.76 (0.62-0.93) **	0.88 (0.72-1.08)	0.87 (0.70-1.07)
Q4	0.62 (0.50-0.76) ***	0.87 (0.70-1.09)	0.83 (0.65-1.05)
	C-Index: 0.559	C-Index: 0.679	C-Index: 0.694
	P-trend < 0.001***	P-trend = 0.37	P for trend = 0.22
WWI (quartiles)			
Q1	1 (Ref)	1 (Ref)	1 (Ref)
Q2	1.49 (1.20-1.85) ***	1.23 (0.99-1.53)	1.28 (1.03-1.60) *
Q3	1.57 (1.26-1.96) ***	1.21 (0.97-1.52)	1.18 (0.95-1.48)
Q4	1.85 (1.49-2.29) ***	1.38 (1.11-1.73) **	1.33 (1.06-1.66) *
	C-Index: 0.559	C-Index: 0.678	C-Index: 0.694
	P-trend < 0.001***	P-trend = 0.007**	P-trend = 0.04*
ABSI (quartiles)			
Q1	1 (Ref)	1 (Ref)	1 (Ref)
Q2	1.43 (1.14-1.79) **	1.20 (0.95-1.52)	1.23 (0.97-1.55)
Q3	1.83 (1.46-2.28) ***	1.39 (1.11-1.75) **	1.40 (1.11-1.77) **
Q4	2.43 (1.96-3.02) ***	1.49 (1.18-1.89) ***	1.43 (1.13-1.82) **
	C-Index: 0.598	C-Index: 0.681	C-Index: 0.697
	P-trend < 0.001***	P-trend < 0.001***	P-trend = 0.002**

P-value: *<0.05, **<0.01, ***<0.001

a Nothing was adjusted.

b Adjusted for age, gender, race.

c Adjusted for age, gender, race, marital, education, drink, smoke, hemoglobin A1c, high-density lipoprotein cholesterol, glucose, total cholesterol, triglyceride.

HR, hazard ration; BMI, body mass index; WWI, weight-adjusted-waist index; ABSI, a body shape index.

**Figure 4:**
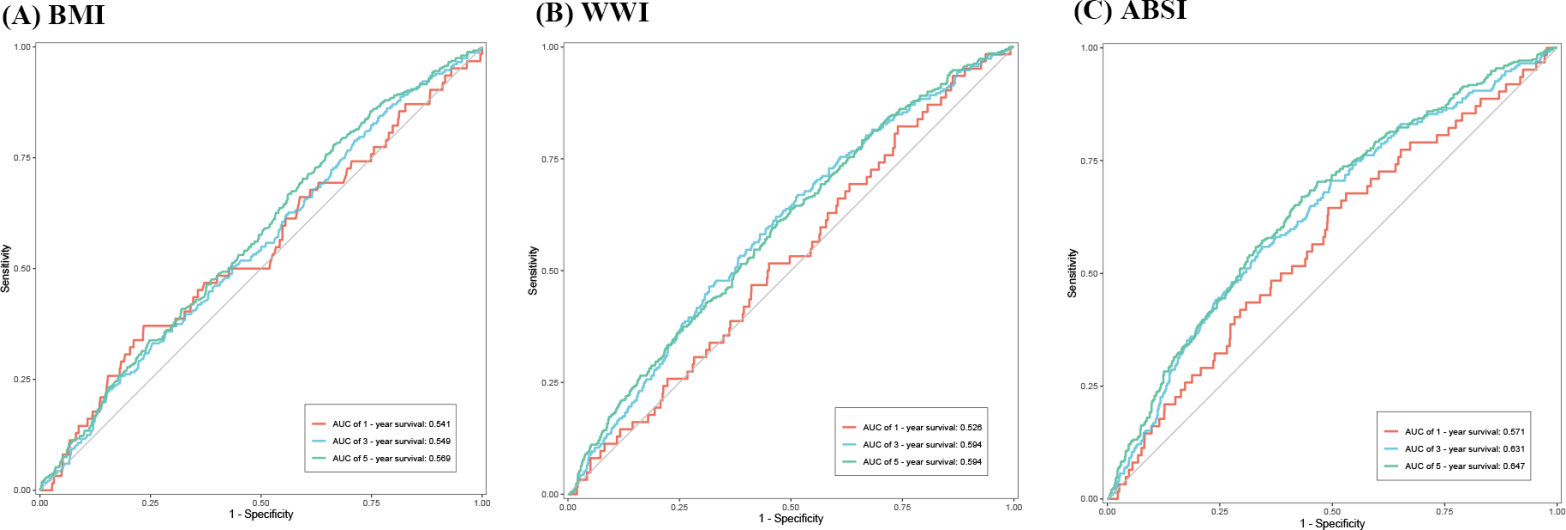
Time-dependent ROC curves indicating the capacity of BMI, WWI, and ABSI to predict Heart Failure (HF) patients’ long-term survival. **(A)** Body mass index (BMI). **(B)** Weight-adjusted-waist index (WWI). **(C)** A body shape index (ABSI). ROC, receiver operating characteristic; AUC, an area under ROC curve.

The time-dependent ROC curves in **Figure 4** show the ability of BMI, WWI, and ABSI to predict HF patients’ 1-, 3-, and 5-year survival. In contrast to BMI, the WWI had an improved ability to predict 3- and 5-year survival (3-year, 0.594 vs 0.549; 5-year, 0.594 vs 0.569). ABSI had a moderate ability to predict survival compared to BMI (1-year 0.571 vs 0.541, 3-year 0.631 vs 0.549, 5-year 0.647 vs 0.569).

### 3.4 Subgroup analysis

Within subgroup analyses, the associations between BMI, WWI, and ABSI and all-cause mortality among HF patients were consistent across most subgroups (**Tables 5**-**7** in **Supplementary Data Sheet 1**). For patients with HF by race, sex, and marital status, WWI and ABSI correlated better than BMI in assessing risk for primary outcomes. The relationship between WWI, ABSI and all-cause mortality was consistent regardless of whether HF patients had comorbid hypertension, CHD, MI, angina and DM, but the correlations were not entirely consistent for BMI. In addition, the results for most subgroups of interactions with BMI, WWI, and ABSI were not statistically significant, except for BMI with hypertension and BMI, ABSI with TC.

## 4 Discussion

Within this cohort study, we compared the association of surrogate adiposity markers with HF prevalence and all-cause mortality and explored the ability of surrogate adiposity markers to predict long-term rates in HF patients. Compared to other markers, we observed that the WWI exhibited a notable correlation with an increased risk of prevalent HF and had good predictive value. In survival analyses of the HF population, the BMI-related “obesity paradox” remained after multivariate adjustment but did not recur with the use of WWI and ABSI. Moreover, the ABSI could better predict long-term survival in HF patients.

All surrogate adiposity markers demonstrated that the greater the level of obesity was, the greater the risk of HF, reaffirming obesity’s independent role as a risk factor in HF development and progression (25, 26). On the one hand, disorders of lipid metabolism caused by obesity destroy the body’s energy homeostasis, resulting in elevated tissue stress and dysfunction (27). Obese patients, especially those with abdominal obesity, often suffer from diabetes, hypertension and other metabolism-related diseases. These chronic diseases are collectively known as MetS (28). Excessive adipocytes lead to a compensatory increase in mitochondrial fatty acid oxidation, leading to increased energy production and generating oxidative stress in adipocytes. This oxidative stress causes adipocytes to express stress markers that are recognized by the body’s immune system, ultimately leading to chronic inflammation (29–31). Besides, metabolic disorders can further cause immune cell activation in the liver, spleen and other tissues, further exacerbating the effects of chronic inflammation on the organism (32). The short-term inflammatory reaction leads to increase immune cells infiltration and expression of pro-inflammatory cytokines in the myocardium, resulting in an increased cardiac load. However, prolonged action of inflammatory cells and cytokines on cardiomyocytes causes left ventricular dysfunction and cardiomyocyte remodeling, which subsequently induces HF (33). On the other hand, obesity induces hemodynamic alterations characterized by elevated blood volume, cardiac output, and blood pressure, linked to the activation of the renin-angiotensin-aldosterone system (RAAS) and heightened sympathetic nerve activity (34, 35). The increasing blood volume leads to an elevated cardiac preload, the long-term effects of which cause ventricular dilatation and myocardial hypertrophy, facilitating the progression of HF (34).

While there exists a notable relationship between overall obesity and abdominal obesity, certain individuals may solely exhibit overall obesity due to fat distribution uniformity (36). Defining obesity based on BMI may omit patients with abdominal obesity, concealing cardiovascular disease risks within this subgroup (37, 38). With the increasingly research on body composition and fat distribution, more and more scholars believe that BMI cannot be used to represent the true fat content (39). A cohort study from the UK Biobank shows that, regardless of BMI, surrogate adiposity markers have the strongest association with mortality (40). Therefore, this study revealed an increased risk of HF when WC and the WHtR were used to evaluate obesity. This may be explained by the fact that WC and WHtR can better describe abdominal obesity. Among the other adiposity markers, the WWI and RFM better reflect the association between obesity and HF. The WWI takes into account differences in fat distribution and skeletal muscle mass among individuals and more accurately reflects abdominal obesity, while the RFM takes into account differences in waist circumference by gender and ethnicity (19, 22). In combination with ROC curves to assess the predictive power of adiposity markers for the prevalence of HF, WWI can be considered a replacement to BMI for the assessment of overweight and obesity.

Similar to the results of other studies exploring the correlation between adiposity markers and all-cause mortality in HF patients (41, 42), this study revealed that a higher BMI was associated with reduced mortality, which is considered the BMI-related “obesity paradox”. However, other studies have expressed different views (14, 43). Therefore, the use of BMI to define overweight or obesity in HF patients is inappropriate. Abdominal obesity is considered a marker of cardiovascular disease risk, including HF (37, 44–46). In this study, WWI and ABSI, which are more highly correlated with abdominal obesity, were selected for survival analysis and prediction of long-term survival in HF patients, and both were superior to BMI. Controversially, the ABSI showed good performance in predicting long-term survival in HF patients, but was not shown to be significantly different in analyses assessing its association with the risk of prevalent HF using the fully adjusted logistic model. This may be due to the fact that the ABSI was also used to predict risk of death when it was originally proposed (17).

Overall, these data suggest that higher degree of abdominal obesity are associated with a higher risk of HF and poorer long-term survival among HF patients. The use of surrogate adiposity markers that are more strongly correlated with abdominal obesity may be a better predictor of HF prevalence and long-term survival. Other studies have also confirmed the possibility of selecting other adiposity markers in patients with HF with reduced ejection fraction (HFrEF) (43). At the same time, exercise and diet should be used to intervene in abdominal obesity to achieve primary prevention of cardiovascular disease. For HF patients, weight control should be carried out along with cardiac rehabilitation to improve the long-term survival rate (47–49).

This study has the following advantages: first, the research sample is drawn from a representative population in the United States. The inclusion of a large number of participants and the long-term follow-up in the study enhance the reliability of the research results. In this study, we for the first time delved into the feasibility of employing surrogate adiposity markers to prognosticate long-term risks within the heart failure population. In addition, the results of this study also support the use of WWI and ABSI to assess obesity in heart failure patients. These findings highlight the clinical significance and application value of surrogate adiposity markers.

However, there are some limitations in this study. First, the present study is an observational study and it is not possible to specify the exact causal relationship. Although multiple methods have been used to adjust for the effects of potential confounders, it is not possible to rule out influences on the study results due to measurement error and unknown effectors. Second, due to limitations of the NHANES database, information on diseases, including HF, was obtained from respondents’ records, and information related to specific medications and hospitalization for HF patients was not available. Therefore, we were unable to base further studies on HF subtypes. Third, as the NHANES survey was exclusively conducted in the US, the generalizability of the results to other populations remains uncertain, warranting future research for validation and broader applicability of the findings. Based on the above issues, more information will be explored in future studies to validate and support our findings.

## 5 Conclusions

After multivariate adjustment, the risk of prevalent HF was better assessed using the WWI. In HF patients, higher WWI and ABSI were linked to a higher all-cause mortality risk, while eliminating the BMI-related “obesity paradox”, highlighting the severe impact of abdominal obesity. Meanwhile, ABSI allow for a better prediction of long-term survival in HF patients. These results suggest that we can redefine overweight and obesity in HF patients with the WWI and ABSI.

## Data Availability

Publicly available datasets were analyzed in this study. This data can be found here: https://www.cdc.gov/nchs/nhanes/index.htm.

## References

[1] Savarese G Becher PM Lund LH Seferovic P Rosano GMC Coats AJS. Global burden of heart failure: a comprehensive and updated review of epidemiology. Cardiovasc Res. (2023) 118:3272–87. doi: 10.1093/cvr/cvac013 35150240

[2] Theresa AM Macro M Marianna A Roy SG Andreas B Michael B. 2021 ESC Guidelines for the diagnosis and treatment of acute and chronic heart failure: Developed by the Task Force for the diagnosis and treatment of acute and chronic heart failure of the European Society of Cardiology (ESC). With the special contribution of the Heart Failure Association (HFA) of the ESC. Eur J Heart failure. (2022) 24(1):4–131. doi: 10.1002/ejhf.2333 35083827

[3] Groenewegen A Rutten FH Mosterd A Hoes AW. Epidemiology of heart failure. Eur J Heart Fail. (2020) 22:1342–56. doi: 10.1002/ejhf.1858 PMC7540043 32483830

[4] Murphy SP Ibrahim NE Januzzi JL. Heart failure with reduced ejection fraction: A review. JAMA. (2020) 324:488–504. doi: 10.1001/jama.2020.10262 32749493

[5] Mamas MA Sperrin M Watson MC Coutts A Wilde K Burton C. Do patients have worse outcomes in heart failure than in cancer? A primary care-based cohort study with 10-year follow-up in Scotland. Eur J Heart Fail. (2017) 19:1095–104. doi: 10.1002/ejhf.822 28470962

[6] Shah KS Xu H Matsouaka RA Bhatt DL Heidenreich PA Hernandez AF. Heart failure with preserved, borderline, and reduced ejection fraction: 5-year outcomes. J Am Coll Cardiol. (2017) 70:2476–86. doi: 10.1016/j.jacc.2017.08.074 29141781

[7] Elmaleh-Sachs A Schwartz JL Bramante CT Nicklas JM Gudzune KA Jay M. Obesity management in adults: A review. JAMA. (2023) 330:2000–15. doi: 10.1001/jama.2023.19897 PMC11325826 38015216

[8] Pan X-F Wang L Pan A. Epidemiology and determinants of obesity in China. Lancet Diabetes Endocrinol. (2021) 9:373–92. doi: 10.1016/S2213-8587(21)00045-0 34022156

[9] Alebna PL Mehta A Yehya A daSilva-deAbreu A Lavie CJ Carbone S. Update on obesity, the obesity paradox, and obesity management in heart failure. Prog Cardiovasc Dis. (2024) 82:34–42. doi: 10.1016/j.pcad.2024.01.003 38199320

[10] Tsujimoto T Kajio H. Abdominal obesity is associated with an increased risk of all-cause mortality in patients with HFpEF. J Am Coll Cardiol. (2017) 70:2739–49. doi: 10.1016/j.jacc.2017.09.1111 29191321

[11] Karlsson T Rask-Andersen M Pan G Höglund J Wadelius C Ek WE. Contribution of genetics to visceral adiposity and its relation to cardiovascular and metabolic disease. Nat Med. (2019) 25:1390–5. doi: 10.1038/s41591-019-0563-7 31501611

[12] Romero-Corral A Somers VK Sierra-Johnson J Thomas RJ Collazo-Clavell ML Korinek J. Accuracy of body mass index in diagnosing obesity in the adult general population. Int J Obes (Lond). (2008) 32:959–66. doi: 10.1038/ijo.2008.11 PMC2877506 18283284

[13] Prillaman M. Why BMI is flawed - and how to redefine obesity. Nature. (2023) 622:232–3. doi: 10.1038/d41586-023-03143-x 37903933

[14] Zhang J Begley A Jackson R Harrison M Pellicori P Clark AL. Body mass index and all-cause mortality in heart failure patients with normal and reduced ventricular ejection fraction: a dose-response meta-analysis. Clin Res Cardiol. (2019) 108:119–32. doi: 10.1007/s00392-018-1302-7 29951802

[15] Ross R Neeland IJ Yamashita S Shai I Seidell J Magni P. Waist circumference as a vital sign in clinical practice: a Consensus Statement from the IAS and ICCR Working Group on Visceral Obesity. Nat Rev Endocrinol. (2020) 16:177–89. doi: 10.1038/s41574-019-0310-7 PMC7027970 32020062

[16] Gelber RP Gaziano JM Orav EJ Manson JE Buring JE Kurth T. Measures of obesity and cardiovascular risk among men and women. J Am Coll Cardiol. (2008) 52:605–15. doi: 10.1016/j.jacc.2008.03.066 PMC2671389 18702962

[17] Krakauer NY Krakauer JC. A new body shape index predicts mortality hazard independently of body mass index. PloS One. (2012) 7:e39504. doi: 10.1371/journal.pone.0039504 22815707 PMC3399847

[18] Park Y Kim NH Kwon TY Kim SG. A novel adiposity index as an integrated predictor of cardiometabolic disease morbidity and mortality. Sci Rep. (2018) 8:16753. doi: 10.1038/s41598-018-35073-4 30425288 PMC6233180

[19] Woolcott OO Bergman RN. Relative fat mass (RFM) as a new estimator of whole-body fat percentage ─ A cross-sectional study in American adult individuals. Sci Rep. (2018) 8:10980. doi: 10.1038/s41598-018-29362-1 30030479 PMC6054651

[20] Kahn HS. The “lipid accumulation product” performs better than the body mass index for recognizing cardiovascular risk: a population-based comparison. BMC Cardiovasc Disord. (2005) 5:26. doi: 10.1186/1471-2261-5-26 16150143 PMC1236917

[21] Amato MC Giordano C Galia M Criscimanna A Vitabile S Midiri M. Visceral Adiposity Index: a reliable indicator of visceral fat function associated with cardiometabolic risk. Diabetes Care. (2010) 33:920–2. doi: 10.2337/dc09-1825 PMC2845052 20067971

[22] Kim KJ Son S Kim KJ Kim SG Kim NH. Weight-adjusted waist as an integrated index for fat, muscle and bone health in adults. J Cachexia Sarcopenia Muscle. (2023) 14:2196–203. doi: 10.1002/jcsm.13302 PMC10570086 37550773

[23] Fang H Xie F Li K Li M Wu Y. Association between weight-adjusted-waist index and risk of cardiovascular diseases in United States adults: a cross-sectional study. BMC Cardiovasc Disord. (2023) 23:435. doi: 10.1186/s12872-023-03452-z 37658325 PMC10474739

[24] Dhana K Kavousi M Ikram MA Tiemeier HW Hofman A Franco OH. Body shape index in comparison with other anthropometric measures in prediction of total and cause-specific mortality. J Epidemiol Community Health. (2016) 70:90–6. doi: 10.1136/jech-2014-205257 26160362

[25] Kenchaiah S Evans JC Levy D Wilson PWF Benjamin EJ Larson MG. Obesity and the risk of heart failure. N Engl J Med. (2002) 347:305–13. doi: 10.1056/NEJMoa020245 12151467

[26] Borlaug BA Jensen MD Kitzman DW Lam CSP Obokata M Rider OJ. Obesity and heart failure with preserved ejection fraction: new insights and pathophysiological targets. Cardiovasc Res. (2023) 118:3434–50. doi: 10.1093/cvr/cvac120 PMC10202444 35880317

[27] Guilherme A Virbasius JV Puri V Czech MP. Adipocyte dysfunctions linking obesity to insulin resistance and type 2 diabetes. Nat Rev Mol Cell Biol. (2008) 9:367–77. doi: 10.1038/nrm2391 PMC2886982 18401346

[28] Després J-P Lemieux I. Abdominal obesity and metabolic syndrome. Nature. (2006) 444:881–7. doi: 10.1038/nature05488 17167477

[29] Bhatti JS Bhatti GK Reddy PH. Mitochondrial dysfunction and oxidative stress in metabolic disorders - A step towards mitochondria based therapeutic strategies. Biochim Biophys Acta Mol Basis Dis. (2017) 1863:1066–77. doi: 10.1016/j.bbadis.2016.11.010 PMC5423868 27836629

[30] Furukawa S Fujita T Shimabukuro M Iwaki M Yamada Y Nakajima Y. Increased oxidative stress in obesity and its impact on metabolic syndrome. J Clin Invest. (2004) 114:1752–61. doi: 10.1172/JCI21625 PMC535065 15599400

[31] Prasun P. Mitochondrial dysfunction in metabolic syndrome. Biochim Biophys Acta Mol Basis Dis. (2020) 1866:165838. doi: 10.1016/j.bbadis.2020.165838 32428560

[32] Andersen CJ Murphy KE Fernandez ML. Impact of obesity and metabolic syndrome on immunity. Adv Nutr. (2016) 7:66–75. doi: 10.3945/an.115.010207 26773015 PMC4717890

[33] Adamo L Rocha-Resende C Prabhu SD Mann DL. Reappraising the role of inflammation in heart failure. Nat Rev Cardiol. (2020) 17:269–85. doi: 10.1038/s41569-019-0315-x 31969688

[34] Alpert MA Omran J Bostick BP. Effects of obesity on cardiovascular hemodynamics, cardiac morphology, and ventricular function. Curr Obes Rep. (2016) 5:424–34. doi: 10.1007/s13679-016-0235-6 27744513

[35] Lastra G Dhuper S Johnson MS Sowers JR. Salt, aldosterone, and insulin resistance: impact on the cardiovascular system. Nat Rev Cardiol. (2010) 7:577–84. doi: 10.1038/nrcardio.2010.123 20697411

[36] Gómez-Ambrosi J Silva C Galofré JC Escalada J Santos S Millán D. Body mass index classification misses subjects with increased cardiometabolic risk factors related to elevated adiposity. Int J Obes (Lond). (2012) 36:286–94. doi: 10.1038/ijo.2011.100 21587201

[37] Piché M-E Poirier P Lemieux I Després J-P. Overview of epidemiology and contribution of obesity and body fat distribution to cardiovascular disease: an update. Prog Cardiovasc Dis. (2018) 61:103–13. doi: 10.1016/j.pcad.2018.06.004 29964067

[38] Sahakyan KR Somers VK Rodriguez-Escudero JP Hodge DO Carter RE Sochor O. Normal-weight central obesity: implications for total and cardiovascular mortality. Ann Intern Med. (2015) 163:827–35. doi: 10.7326/M14-2525 PMC4995595 26551006

[39] Lee SW Son JY Kim JM Hwang S-S Han JS Heo NJ. Body fat distribution is more predictive of all-cause mortality than overall adiposity. Diabetes Obes Metab. (2018) 20:141–7. doi: 10.1111/dom.13050 28671751

[40] Khan I Chong M Le A Mohammadi-Shemirani P Morton R Brinza C. Surrogate adiposity markers and mortality. JAMA Netw Open. (2023) 6:e2334836. doi: 10.1001/jamanetworkopen.2023.34836 37728925 PMC10512100

[41] Mahajan R Stokes M Elliott A Munawar DA Khokhar KB Thiyagarajah A. Complex interaction of obesity, intentional weight loss and heart failure: a systematic review and meta-analysis. Heart. (2020) 106:58–68. doi: 10.1136/heartjnl-2019-314770 31530572

[42] Horwich TB Fonarow GC Clark AL. Obesity and the obesity paradox in heart failure. Prog Cardiovasc Dis. (2018) 61:151–6. doi: 10.1016/j.pcad.2018.05.005 29852198

[43] Butt JH Petrie MC Jhund PS Sattar N Desai AS Køber L. Anthropometric measures and adverse outcomes in heart failure with reduced ejection fraction: revisiting the obesity paradox. Eur Heart J. (2023) 44:1136–53. doi: 10.1093/eurheartj/ehad083 PMC10111968 36944496

[44] Powell-Wiley TM Poirier P Burke LE Després J-P Gordon-Larsen P Lavie CJ. Obesity and cardiovascular disease: A scientific statement from the american heart association. Circulation. (2021) 143:e984–e1010. doi: 10.1161/CIR.0000000000000973 33882682 PMC8493650

[45] Abraham TM Pedley A Massaro JM Hoffmann U Fox CS. Association between visceral and subcutaneous adipose depots and incident cardiovascular disease risk factors. Circulation. (2015) 132:1639–47. doi: 10.1161/CIRCULATIONAHA.114.015000 PMC4779497 26294660

[46] Shah RV Murthy VL Abbasi SA Blankstein R Kwong RY Goldfine AB. Visceral adiposity and the risk of metabolic syndrome across body mass index: the MESA Study. JACC Cardiovasc Imaging. (2014) 7(12):1221–35. doi: 10.1016/j.jcmg.2014.07.017 PMC4268163 25440591

[47] Bozkurt B Fonarow GC Goldberg LR Guglin M Josephson RA Forman DE. Cardiac rehabilitation for patients with heart failure: JACC expert panel. J Am Coll Cardiol. (2021) 77:1454–69. doi: 10.1016/j.jacc.2021.01.030 33736829

[48] Del Buono MG Arena R Borlaug BA Carbone S Canada JM Kirkman DL. Exercise intolerance in patients with heart failure: JACC state-of-the-art review. J Am Coll Cardiol. (2019) 73:2209–25. doi: 10.1016/j.jacc.2019.01.072 31047010

[49] Kosiborod MN Abildstrøm SZ Borlaug BA Butler J Rasmussen S Davies M. Semaglutide in patients with heart failure with preserved ejection fraction and obesity. N Engl J Med. (2023) 389:1069–84. doi: 10.1056/NEJMoa2306963 37622681

