# Supplementary material for: Association of surrogate adiposity markers with prevalence, all-cause mortality and long-term survival of heart failure: a retrospective study from NHANES database
Source: Front Endocrinol (Lausanne). 2025 Mar 4;16:1430277. doi: 10.3389/fendo.2025.1430277 (PMC11913658; doi:10.3389/fendo.2025.1430277)
Supplement: Supplementary file 1 [file DataSheet1.docx]

**Supplementary Material**

**Table 1:** The formulas of surrogate adiposity markers.

**Table 2:** Baseline characteristics of the study participants based on the reported Heart Failure (HF).

**Table 3:** Association of surrogate adiposity markers with the prevalence of Heart Failure (HF).

**Table 4:** Effect of standardized surrogate adiposity markers level on survival in HF patients: HR from segmented Cox regression analysis based on the inflection point of RCS curves.

**Table 5:** Subgroup analysis of BMI and All-cause mortality in HF patients.

**Table 6:** Subgroup analysis of WWI and All-cause mortality in HF patients.

**Table 7:** Subgroup analysis of ABSI and All-cause mortality in HF patients.

**Figure 1:** Flowchart for study population selection.

**Figure 2:** K-M survival analysis for all-cause mortality in HF patients.

**Figure 3:** K-M survival analysis for all-cause mortality in HF patients. (Surrogate adiposity markers without significant differences)

**Table 1: The formulas of surrogate adiposity markers.**

| Categories | Definition |
| --- | --- |
| BMI | height (m) / weight^2^ |
| WHtR | WC / height (cm) |
| WWI | WC / weight^0.5^ |
| ABSI | (WC/100) × weight^2/3^ × height (m)^5/6^ |
| VAI | Males: WC/(39.68 + 1.88×BMI) × TG/1.03 ×1.31/HDL-C  Females: WC/(36.58 + 1.89×BMI) × TG/0.81 ×1.52/HDL-C |
| LAP | Males: (WC – 65) × TG  Females: (WC – 58) × TG |
| RFM | Males: 64 - (20×(height (cm) / WC))  Females: 76 - (20×(height (cm) / WC)) |

Abbreviations: Weight (kg); BMI, body mass index (kg/m^2^); WC, waist circumference (cm); HDL-C, high-density lipoprotein cholesterol (mmol/L); TG, triglyceride (mmol/L); WHtR, waist-to-height ratio; WWI, weight-adjusted-waist index; ABSI, a body shape index; LAP, lipid accumulation product; VAI, visceral fat index; RFM, relative fat mass.

**Table 2: Baseline characteristics of the study participants based on the reported Heart Failure (HF).**

| Characteristics | Overall  (N=46257) | HF  (N=1366) | Non-HF  (N=44891) | P-value |
| --- | --- | --- | --- | --- |
| **Demographic** |  |  |  |  |
| Age, years, mean (SD) | 46.76 (16.71) | 65.53 (13.39) | 46.35 (16.54) | <0.001 |
| Gender, n (%) |  |  |  |  |
| Male | 22378 (48.4) | 787 (54.2) | 21591 (48.3) | 0.001 |
| Female | 23879 (51.6) | 579 (45.8) | 23300 (51.7) |  |
| Race, n (%) |  |  |  |  |
| Mexican American | 8296 (8.3) | 155 (4.1) | 8141 (8.3) | <0.001 |
| Other Hispanic | 3850 (5.6) | 90 (4.5) | 3760 (5.7) |  |
| Non-Hispanic White | 20671 (68.9) | 750 (73.8) | 19921 (68.7) |  |
| Non-Hispanic Black | 9236 (10.5) | 305 (13.0) | 8931 (10.4) |  |
| Other Race | 4204 (6.8) | 66 (4.7) | 4138 (6.8) |  |
| Marital, n (%) |  |  |  |  |
| Married | 28026 (63.8) | 713 (56.1) | 27313 (63.9) | <0.001 |
| Divorced | 6207 (12.3) | 231 (15.5) | 5976 (12.3) |  |
| Widowed | 3615 (5.5) | 306 (20.2) | 3309 (5.2) |  |
| Single | 7956 (17.3) | 102 (6.9) | 7854 (17.5) |  |
| Others | 453 (1.1) | 14 (1.2) | 439 (1.1) |  |
| Education, n (%) |  |  |  |  |
| Below high school | 12259 (16.9) | 540 (32.1) | 11719 (16.6) | <0.001 |
| High School or GED | 10679 (23.9) | 342 (27.4) | 10377 (23.8) |  |
| Above High School | 23273 (59.1) | 482 (40.4) | 22791 (59.5) |  |
| Others | 46 (0.1) | 2 (0.1) | 44 (0.1) |  |
| **Life-style** |  |  |  |  |
| Smoke, n (%) |  |  |  |  |
| Never | 25164 (53.7) | 517 (36.4) | 24647 (54.1) | <0.001 |
| Ever | 11414 (24.8) | 576 (42.6) | 10838 (24.5) |  |
| Current | 9679 (21.5) | 273 (21.0) | 9406 (21.5) |  |
| Drink, n (%) |  |  |  |  |
| No | 11860 (21.2) | 412 (30.6) | 11448 (21.0) | <0.001 |
| Yes | 31508 (73.4) | 895 (65.9) | 30613 (73.5) |  |
| Others | 2889 (5.5) | 59 (3.5) | 2830 (5.5) |  |
| **Laboratory** |  |  |  |  |
| Glucose, mg/dL, median (IQR) | 91.00  [84.00, 100.00] | 100.00  [90.00, 125.00] | 91.00  [84.00, 100.00] | <0.001 |
| HbA1c, %, median (IQR) | 5.40 [5.10, 5.70] | 5.80 [5.40, 6.50] | 5.40 [5.10, 5.70] | <0.001 |
| TC, mg/dL, median (IQR) | 193.00  [168.00, 221.00] | 179.00  [149.00, 211.00] | 194.00  [168.00, 222.00] | <0.001 |
| TG, mg/dL, median (IQR) | 118.00  [79.00, 180.00] | 140.00  [98.00, 205.77] | 117.00  [79.00, 180.00] | <0.001 |
| HDL-C, mg/dL, median (IQR) | 51.00 [42.00, 62.00] | 45.00 [38.00, 56.00] | 51.00 [42.00, 62.00] | <0.001 |
| **Surrogate adiposity markers** |  |  |  |  |
| BMI, median (IQR) | 27.63 [24.05, 32.07] | 30.20 [26.16, 35.68] | 27.60 [24.00, 32.00] | <0.001 |
| WC, median (IQR) | 97.00  [86.60, 108.20] | 107.20  [96.60, 119.36] | 96.90  [86.50, 107.90] | <0.001 |
| WHtR, median (IQR) | 0.57 [0.51, 0.64] | 0.64 [0.58, 0.71] | 0.57 [0.51, 0.64] | <0.001 |
| WWI, median (IQR) | 10.89 [10.34, 11.47] | 11.59 [11.10, 12.15] | 10.87 [10.33, 11.45] | <0.001 |
| ABSI, median (IQR) | 0.0812  [0.0780, 0.0845] | 0.0851  [0.0818, 0.0880] | 0.0811  [0.0779, 0.0843] | <0.001 |
| LAP, median (IQR) | 47.81 [25.12, 86.24] | 74.13  [41.83, 121.82] | 47.28 [24.88, 85.23] | <0.001 |
| VAI, median (IQR) | 1.66 [0.98, 2.91] | 2.30 [1.33, 3.77] | 1.65 [0.97, 2.89] | <0.001 |
| RFM, median (IQR) | 34.39 [28.79, 41.83] | 36.95 [31.52, 44.94] | 34.32 [28.72, 41.78] | <0.001 |
| **Diseases** |  |  |  |  |
| Hypertension, n, % | 20514 (39.5) | 1141 (80.4) | 19373 (38.6) | <0.001 |
| CHD, n, % | 1837 (3.4) | 564 (40.9) | 1273 (2.5) | <0.001 |
| MI, n, % | 1897 (3.2) | 607 (44.0) | 1290 (2.3) | <0.001 |
| Angina, n, % | 1274 (2.3) | 355 (27.1) | 919 (1.8) | <0.001 |
| DM, n, % | 6858 (10.8) | 585 (38.8) | 6273 (10.2) | <0.001 |
| **Events** |  |  |  |  |
| All-cause mortality, n, % | 6609 (10.4) | 700 (47.8) | 5909 (9.6) | <0.001 |

Abbreviations:

SD, standard deviation; IQR, interquartile range.

Laboratory: HbA1c, hemoglobin A1c; TC, total cholesterol; TG, triglyceride; HDL-C, high-density lipoprotein cholesterol.

Examination: BMI, body mass index; WC, waist circumference; WHtR, waist-to-height ratio; WWI, weight-adjusted-waist index; ABSI, a body shape index; LAP, lipid accumulation product; VAI, visceral fat index; RFM, relative fat mass.

Diseases: CHD, coronary heart disease; MI, myocardial infarction; DM, diabetes mellitus.

**Table 3: Association of surrogate adiposity markers with the prevalence of Heart Failure (HF).**

| Characteristics | Model 1^a^  OR (95%CI), P-value | Model 2^b^  OR (95%CI), P-value | Model 3^c^  OR (95%CI), P-value |
| --- | --- | --- | --- |
| BMI (quartiles) |  |  |  |
| Quartile 1 | 1 (Reference) | 1 (Reference) | 1 (Reference) |
| Quartile 2 | 1.31 (1.05-1.63), 0.02* | 1.05 (0.84-1.31), 0.67 | 1.03 (0.83-1.28), 0.80 |
| Quartile 3 | 1.85 (1.48-2.32), <0.001*** | 1.47 (1.17-1.85), 0.001** | 1.34 (1.06-1.69), 0.01* |
| Quartile 4 | 2.74 (2.16-3.48), <0.001*** | 2.77 (2.14-3.58), <0.001*** | 2.21 (1.71-2.85), <0.001*** |
|  | P for trend < 0.001*** | P for trend < 0.001*** | P for trend < 0.001*** |
| WC (quartiles) |  |  |  |
| Quartile 1 | 1 (Reference) | 1 (Reference) | 1 (Reference) |
| Quartile 2 | 1.65 (1.26-2.15), <0.001*** | 1.13 (0.87-1.46), 0.36 | 1.07 (0.83-1.38), 0.60 |
| Quartile 3 | 2.90 (2.28-3.69), <0.001*** | 1.64 (1.28-2.11), <0.001*** | 1.45 (1.12-1.88), 0.005** |
| Quartile 4 | 5.06 (3.96-6.48), <0.001*** | 3.09 (2.39-4.00), <0.001*** | 2.32 (1.78-3.04), <0.001*** |
|  | P for trend < 0.001*** | P for trend < 0.001*** | P for trend < 0.001*** |
| WHtR (quartiles) |  |  |  |
| Quartile 1 | 1 (Reference) | 1 (Reference) | 1 (Reference) |
| Quartile 2 | 2.07 (1.53-2.81), <0.001*** | 1.22 (0.89-1.67), 0.21 | 1.18 (0.86-1.64), 0.30 |
| Quartile 3 | 3.90 (3.03-5.02), <0.001*** | 1.90 (1.46-2.47), <0.001*** | 1.68 (1.26-2.23), <0.001*** |
| Quartile 4 | 6.35 (4.85-8.31), <0.001*** | 3.28 (2.49-4.32), <0.001*** | 2.44 (1.82-3.28), <0.001*** |
|  | P for trend < 0.001*** | P for trend < 0.001*** | P for trend < 0.001*** |
| WWI (quartiles) |  |  |  |
| Quartile 1 | 1 (Reference) | 1 (Reference) | 1 (Reference) |
| Quartile 2 | 2.53 (1.85-3.44), <0.001*** | 1.53 (1.12-2.08), 0.008** | 1.42 (1.04-1.93), 0.03* |
| Quartile 3 | 5.66 (4.32-7.41), <0.001*** | 2.38 (1.80-3.17), <0.001*** | 1.91 (1.43-2.53), <0.001*** |
| Quartile 4 | 11.15 (8.59-14.48), <0.001*** | 3.58 (2.72-4.70), <0.001*** | 2.37 (1.79-3.14), <0.001*** |
|  | P for trend < 0.001*** | P for trend < 0.001*** | P for trend < 0.001*** |
| ABSI (quartiles) |  |  |  |
| Quartile 1 | 1 (Reference) | 1 (Reference) | 1 (Reference) |
| Quartile 2 | 1.42 (1.04-1.93), 0.03* | 0.96 (0.68-1.32), 0.79 | 0.90 (0.65-1.24), 0.51 |
| Quartile 3 | 2.74 (2.05-3.66), <0.001*** | 1.24 (0.90-1.71), 0.18 | 1.06 (0.77-1.47), 0.71 |
| Quartile 4 | 6.52 (5.04-8.53), <0.001*** | 1.68 (1.23-2.30), 0.001** | 1.22 (0.88-1.68), 0.23 |
|  | P for trend < 0.001*** | P for trend < 0.001*** | P for trend = 0.04 |
| LAP (quartiles) |  |  |  |
| Quartile 1 | 1 (Reference) | 1 (Reference) | 1 (Reference) |
| Quartile 2 | 1.98 (1.59-2.50), <0.001*** | 1.25 (0.99-1.57), 0.06 | 1.26 (0.99-1.59), 0.06 |
| Quartile 3 | 2.54 (2.00-3.23), <0.001*** | 1.47 (1.14-1.88), 0.003** | 1.43 (1.09-1.88), 0.01* |
| Quartile 4 | 4.09 (3.19-5.26), <0.001*** | 2.65 (2.05-3.42), <0.001*** | 2.62 (1.83-3.75), <0.001*** |
|  | P for trend < 0.001*** | P for trend < 0.001*** | P for trend < 0.001*** |
| VAI (quartiles) |  |  |  |
| Quartile 1 | 1 (Reference) | 1 (Reference) | 1 (Reference) |
| Quartile 2 | 1.35 (1.05-1.74), 0.0189* | 1.23 (0.95-1.59), 0.12 | 1.02 (0.77-1.35), 0.89 |
| Quartile 3 | 2.00 (1.56-2.57), <0.001*** | 1.71 (1.33-2.22), <0.001*** | 1.23 (0.91-1.66), 0.17 |
| Quartile 4 | 2.48 (1.97-3.12), <0.001*** | 2.19 (1.73-2.79), <0.001*** | 1.29 (0.86-1.94), 0.21 |
|  | P for trend < 0.001*** | P for trend < 0.001*** | P for trend = 0.12 |
| RFM (quartiles) |  |  |  |
| Quartile 1 | 1 (Reference) | 1 (Reference) | 1 (Reference) |
| Quartile 2 | 2.40 (1.92-2.99), <0.001*** | 1.90 (1.51-2.38), <0.001*** | 1.68 (1.32-2.12), <0.001*** |
| Quartile 3 | 2.02 (1.56-2.62), <0.001*** | 3.73 (2.79-4.99), <0.001*** | 2.80 (2.08-3.77), <0.001*** |
| Quartile 4 | 3.16 (2.51-3.98), <0.001*** | 8.20 (5.63-11.95), <0.001*** | 4.98 (3.41-7.26), <0.001*** |
|  | P for trend < 0.001*** | P for trend < 0.001*** | P for trend < 0.001*** |

P-value: * <0.05, ** <0.01, *** <0.001

^a^ Model 1: Nothing was adjusted.

^b^ Model 2: Adjusted for age, gender, race.

^c^ Model 3: Adjusted for age, gender, race, marital, education, drink, smoke, hemoglobin A1c, high-density lipoprotein cholesterol, glucose, total cholesterol, triglyceride.

Abbreviations: OR, odds ratio; BMI, body mass index; WC, waist circumference; WHtR, waist-to-height ratio; WWI, weight-adjusted-waist index; ABSI, a body shape index; LAP, lipid accumulation product; VAI, visceral fat index; RFM, relative fat mass.

**Table 4: Effect of standardized surrogate adiposity markers level on survival in HF patients: HR from segmented Cox regression analysis based on the inflection point of RCS curves.**

| Characteristics | HR per SD (95% CI) | P-value |
| --- | --- | --- |
| BMI |  |  |
| < 29.76 | 0.86 (0.78-0.95) | 0.003** |
| ≥29.76 | 0.86 (0.76-0.97) | 0.015* |
| WWI |  |  |
| < 10.63 | 1.17 (0.88-1.55) | 0.29 |
| ≥10.63 | 1.16 (1.07-1.25) | <0.001*** |
| ABSI |  |  |
| <0.0785 | 1.22 (0.90-1.64) | 0.20 |
| ≥0.0785 | 1.31 (1.22-1.41) | <0.001*** |

P-value: * <0.05, ** <0.01, *** <0.001

Abbreviations: RCS, restricted cubic spline; HF, heart failure; HR, hazard ratios; SD, standard deviation; BMI, body mass index; WWI, weight-adjusted-waist index; ABSI, a body shape index.

**Table 5: Subgroup analysis of BMI and All-cause mortality in HF patients.**

| Subgroups | Case | Total | Q1 | Q2 HR (95%) | Q3 HR (95%) | Q4 HR (95%) | P for interaction |
| --- | --- | --- | --- | --- | --- | --- | --- |
| Age，years |  |  |  |  |  |  |  |
| ≤54 | 47 | 219 | Ref | 0.52 (0.21-1.33) | 0.89 (0.37-2.01) | 1.24 (0.60-2.59) | 0.768 |
| 55-64 | 114 | 274 | Ref | 0.81 (0.46-1.43) | 0.96 (0.55-1.66) | 0.98 (0.58-1.64) |  |
| 65-74 | 204 | 402 | Ref | 0.66 (0.44-0.99) * | 0.75 (0.51-1.11) | 0.70 (0.48-1.04) |  |
| ≥75 | 335 | 471 | Ref | 0.78 (0.60-1.01) | 0.88 (0.66-1.17) | 0.72 (0.49-1.07) |  |
| Gender |  |  |  |  |  |  |  |
| Male | 415 | 787 | Ref | 0.81 (0.63-1.04) | 0.79 (0.61-1.03) | 0.75 (0.56-1.01) | 0.257 |
| Female | 285 | 579 | Ref | 0.61 (0.44-0.84) ** | 0.70 (0.51-0.98) * | 0.50 (0.36-0.68) *** |  |
| Race |  |  |  |  |  |  |  |
| Mexican American | 83 | 155 | Ref | 0.48 (0.26-0.87) * | 0.67 (0.39-1.14) | 0.54 (0.27-1.06) | 0.922 |
| Other Hispanic | 25 | 90 | Ref | 0.62 (0.18-2.06) | 0.88 (0.26-3.03) | 0.74 (0.20-2.77) |  |
| Non-Hispanic White | 448 | 750 | Ref | 0.75 (0.59-0.96) * | 0.80 (0.62-1.03) | 0.61 (0.47-0.81) *** |  |
| Non-Hispanic Black | 124 | 305 | Ref | 0.85 (0.51-1.42) | 0.68 (0.41-1.14) | 0.63 (0.39-1.01) |  |
| Other Race | 20 | 66 | Ref | 0.90 (0.24-3.42) | 1.11 (0.33-3.69) | 1.44 (0.47-4.44) |  |
| Marital |  |  |  |  |  |  |  |
| Married | 347 | 713 | Ref | 0.76 (0.57-1.01) | 0.82 (0.62-1.09) | 0.63 (0.46-0.86) ** | 0.938 |
| Other marital ^a^ | 353 | 653 | Ref | 0.72 (0.54-0.95) * | 0.72 (0.54-0.97) * | 0.60 (0.45-0.81) *** |  |
| Hypertension |  |  |  |  |  |  |  |
| Yes | 571 | 1141 | Ref | 0.67 (0.54-0.84) *** | 0.70 (0.56-0.87) ** | 0.52 (0.41-0.66) *** | 0.004 |
| No | 129 | 225 | Ref | 0.97 (0.62-1.51) | 1.02 (0.64-1.65) | 1.44 (0.87-2.39) |  |
| CHD |  |  |  |  |  |  |  |
| Yes | 297 | 564 | Ref | 0.69 (0.51-0.94) * | 0.81 (0.60-1.09) | 0.71 (0.50-1.00) | 0.556 |
| No | 403 | 802 | Ref | 0.76 (0.59-0.99) * | 0.71 (0.54-0.94) * | 0.58 (0.44-0.76) *** |  |
| MI |  |  |  |  |  |  |  |
| Yes | 324 | 607 | Ref | 0.75 (0.56-1.00) * | 0.82 (0.61-1.10) | 0.69 (0.50-0.96) * | 0.809 |
| No | 376 | 759 | Ref | 0.72 (0.55-0.95) * | 0.71 (0.54-0.95) * | 0.57 (0.43-0.76) *** |  |
| Angina |  |  |  |  |  |  |  |
| Yes | 196 | 355 | Ref | 0.72 (0.49-1.05) | 0.77 (0.53-1.12) | 0.63 (0.42-0.97) * | 0.997 |
| No | 504 | 1011 | Ref | 0.74 (0.58-0.93) * | 0.76 (0.59-0.97) * | 0.61 (0.48-0.78) *** |  |
| DM |  |  |  |  |  |  |  |
| Yes | 315 | 585 | Ref | 0.73 (0.51-1.03) | 0.76 (0.54-1.06) | 0.60 (0.43-0.84) ** | 0.773 |
| No | 385 | 781 | Ref | 0.70 (0.55-0.89) ** | 0.65 (0.50-0.86) ** | 0.49 (0.35-0.68) *** |  |
| TC |  |  |  |  |  |  |  |
| >200mg/dl | 213 | 405 | Ref | 0.58 (0.41-0.83) ** | 0.55 (0.37-0.80) ** | 0.62 (0.42-0.90) * | 0.750 |
| ≤200mg/dl | 487 | 961 | Ref | 0.82 (0.65-1.04) | 0.87 (0.69-1.11) | 0.61 (0.47-0.79) *** |  |
| TG |  |  |  |  |  |  |  |
| >150mg/dl | 291 | 592 | Ref | 0.70 (0.50-0.96) * | 0.65 (0.46-0.90) * | 0.61 (0.43-0.85) ** | 0.043 |
| ≤150mg/dl | 409 | 774 | Ref | 0.77 (0.60-0.99) * | 0.88 (0.68-1.14) | 0.63 (0.47-0.84) ** |  |

P-value: * <0.05, ** <0.01, *** <0.001

Abbreviations: BMI, body mass index; HF, heart failure; CHD, coronary heart disease; MI, myocardial infarction; DM, diabetes mellitus.TC, total cholesterol; TG, triglyceride.

^a^ The other marital includes divorced, widowed, single, and others.

**Table 6: Subgroup analysis of WWI and All-cause mortality in HF patients.**

| Subgroups | Case | Total | Q1 | Q2 HR (95%) | Q3 HR (95%) | Q4 HR (95%) | P for interaction |
| --- | --- | --- | --- | --- | --- | --- | --- |
| Age，years |  |  |  |  |  |  |  |
| ≤54 | 47 | 219 | Ref | 1.84 (0.92-3.69) | 1.39 (0.58-3.37) | 2.49 (1.07-5.83) * | 0.822 |
| 55-64 | 114 | 274 | Ref | 1.33 (0.80-2.23) | 1.21 (0.73-2.01) | 1.48 (0.86-2.55) |  |
| 65-74 | 204 | 402 | Ref | 1.17 (0.79-1.72) | 1.30 (0.86-1.96) | 1.14 (0.77-1.70) |  |
| ≥75 | 335 | 471 | Ref | 1.21 (0.86-1.69) | 1.12 (0.81-1.56) | 1.30 (0.95-1.80) |  |
| Gender |  |  |  |  |  |  |  |
| Male | 415 | 787 | Ref | 1.44 (1.10-1.89) ** | 1.78 (1.35-2.34) *** | 2.13 (1.58-2.86) *** | 0.210 |
| Female | 285 | 579 | Ref | 1.59 (1.11-2.27) * | 1.28 (0.89-1.84) | 1.70 (1.23-2.35) ** |  |
| Race |  |  |  |  |  |  |  |
| Mexican American | 83 | 155 | Ref | 2.32 (1.14-4.71) * | 2.29 (1.10-4.78) * | 2.17 (1.10-4.26) * | 0.231 |
| Other Hispanic | 25 | 90 | Ref | 1.47 (0.41-5.27) | 1.20 (0.36-3.98) | 2.42 (0.79-7.40) |  |
| Non-Hispanic White | 448 | 750 | Ref | 1.23 (0.93-1.62) | 1.43 (1.09-1.87) * | 1.76 (1.34-2.31) *** |  |
| Non-Hispanic Black | 124 | 305 | Ref | 2.00 (1.29-3.11) ** | 1.30 (0.75-2.23) | 1.72 (1.02-2.89) * |  |
| Other Race | 20 | 66 | Ref | 1.57 (0.39-6.32) | 3.92 (0.96-15.99) | 1.98 (0.47-8.34) |  |
| Marital |  |  |  |  |  |  |  |
| Married | 347 | 713 | Ref | 1.45 (1.08-1.94) * | 1.51 (1.11-2.05) ** | 1.81 (1.30-2.51) *** | 0.911 |
| Other marital ^a^ | 353 | 653 | Ref | 1.64 (1.18-2.27) ** | 1.66 (1.22-2.27) ** | 1.81 (1.36-2.42) *** |  |
| Hypertension |  |  |  |  |  |  |  |
| Yes | 571 | 1141 | Ref | 1.43 (1.12-1.83) ** | 1.49 (1.17-1.90) ** | 1.81 (1.43-2.30) *** | 0.804 |
| No | 129 | 225 | Ref | 1.74 (1.10-2.74) * | 2.14 (1.28-3.56) ** | 2.04 (1.21-3.43) ** |  |
| CHD |  |  |  |  |  |  |  |
| Yes | 297 | 564 | Ref | 1.53 (1.10-2.14) * | 1.51 (1.08-2.11) * | 1.69 (1.21-2.38) ** | 0.839 |
| No | 403 | 802 | Ref | 1.44 (1.09-1.91) * | 1.60 (1.20-2.14) ** | 1.94 (1.47-2.56) *** |  |
| MI |  |  |  |  |  |  |  |
| Yes | 324 | 607 | Ref | 1.59 (1.16-2.19) ** | 1.82 (1.31-2.53) *** | 1.69 (1.22-2.36) ** | 0.190 |
| No | 376 | 759 | Ref | 1.40 (1.04-1.87) * | 1.39 (1.04-1.87) * | 1.99 (1.50-2.64) *** |  |
| Angina |  |  |  |  |  |  |  |
| Yes | 196 | 355 | Ref | 1.88 (1.23-2.88) ** | 1.89 (1.23-2.91) ** | 2.19 (1.43-3.36) *** | 0.722 |
| No | 504 | 1011 | Ref | 1.37 (1.07-1.76) * | 1.48 (1.14-1.90) ** | 1.75 (1.36-2.24) *** |  |
| DM |  |  |  |  |  |  |  |
| Yes | 315 | 585 | Ref | 1.50 (1.06-2.13) * | 1.31 (0.92-1.85) | 1.50 (1.07-2.12) * | 0.233 |
| No | 385 | 781 | Ref | 1.38 (1.04-1.82) * | 1.67 (1.25-2.22) *** | 1.98 (1.49-2.61) *** |  |
| TC |  |  |  |  |  |  |  |
| >200mg/dl | 213 | 405 | Ref | 1.28 (0.88-1.88) | 1.43 (0.97-2.11) | 1.57 (1.07-2.31) * | 0.115 |
| ≤200mg/dl | 487 | 961 | Ref | 1.60 (1.23-2.07) *** | 1.65 (1.27-2.15) *** | 1.99 (1.54-2.58) *** |  |
| TG |  |  |  |  |  |  |  |
| >150mg/dl | 291 | 592 | Ref | 1.12 (0.79-1.59) | 1.12 (0.79-1.59) | 1.51 (1.07-2.12) * | 0.527 |
| ≤150mg/dl | 409 | 774 | Ref | 1.80 (1.37-2.38) *** | 2.05 (1.55-2.72) *** | 2.17 (1.64-2.87) *** |  |

P-value: * <0.05, ** <0.01, *** <0.001

Abbreviations: WWI, weight-adjusted-waist index; HF, heart failure; CHD, coronary heart disease; MI, myocardial infarction; DM, diabetes mellitus.TC, total cholesterol; TG, triglyceride.

^a^ The other marital includes divorced, widowed, single, and others.

**Table 7: Subgroup analysis of ABSI and All-cause mortality in HF patients.**

| Subgroups | Case | Total | Q1 | Q2 HR (95%) | Q3 HR (95%) | Q4 HR (95%) | P for interaction | |
| --- | --- | --- | --- | --- | --- | --- | --- | --- |
| Age，years |  |  |  |  |  |  |  |  |
| ≤54 | 47 | 219 | Ref | 1.28 (0.61-2.67) | 1.94 (0.89-4.24) | 3.65 (1.57-8.50) ** | 0. 299 |  |
| 55-64 | 114 | 274 | Ref | 1.03 (0.61-1.74) | 1.45 (0.88-2.40) | 1.96 (1.12-3.43) * |  |  |
| 65-74 | 204 | 402 | Ref | 1.32 (0.86-2.01) | 1.77 (1.18-2.65) ** | 1.52 (0.99-2.32) |  |  |
| ≥75 | 335 | 471 | Ref | 1.31 (0.92-1.86) | 1.23 (0.86-1.74) | 1.44 (1.05-1.99) * |  |  |
| Gender |  |  |  |  |  |  |  |  |
| Male | 415 | 787 | Ref | 1.53 (1.08-2.18) * | 2.15 (1.53-3.01) *** | 2.89 (2.07-4.05) *** | 0. 315 |  |
| Female | 285 | 579 | Ref | 1.47 (1.07-2.01) * | 1.60 (1.15-2.22) ** | 2.07 (1.51-2.84) *** |  |  |
| Race |  |  |  |  |  |  |  |  |
| Mexican American | 83 | 155 | Ref | 2.16 (1.12-4.16) * | 1.76 (0.92-3.38) | 2.08 (1.07-4.05) * | 0. 329 |  |
| Other Hispanic | 25 | 90 | Ref | 1.25 (0.33-4.72) | 5.72 (1.85-17.65) ** | 2.65 (0.81-8.73) |  |  |
| Non-Hispanic White | 448 | 750 | Ref | 1.15 (0.85-1.56) | 1.45 (1.08-1.96) * | 1.98 (1.48-2.63) *** |  |  |
| Non-Hispanic Black | 124 | 305 | Ref | 1.43 (0.91-2.25) | 1.96 (1.22-3.17) ** | 2.78 (1.58-4.89) *** |  |  |
| Other Race | 20 | 66 | Ref | 2.40 (0.44-13.10) | 4.62 (1.00-21.44) | 4.89 (0.94-25.36) |  |  |
| Marital |  |  |  |  |  |  |  |  |
| Married | 347 | 713 | Ref | 1.41 (1.02-1.96) * | 1.86 (1.34-2.57) *** | 2.65 (1.91-3.67) *** | 0. 692 |  |
| Other marital ^a^ | 353 | 653 | Ref | 1.56 (1.14-2.14) ** | 1.91 (1.40-2.59) *** | 2.30 (1.72-3.08) *** |  |  |
| Hypertension |  |  |  |  |  |  |  |  |
| Yes | 571 | 1141 | Ref | 1.50 (1.16-1.93) ** | 1.85 (1.44-2.37) *** | 2.54 (2.00-3.24) *** | 0. 734 |  |
| No | 129 | 225 | Ref | 1.18 (0.71-1.95) | 1.85 (1.12-3.05) * | 1.98 (1.20-3.28) ** |  |  |
| CHD |  |  |  |  |  |  |  |  |
| Yes | 297 | 564 | Ref | 1.58 (1.08-2.29) * | 2.06 (1.43-2.96) *** | 2.18 (1.51-3.14) *** | 0. 175 |  |
| No | 403 | 802 | Ref | 1.33 (0.99-1.77) | 1.66 (1.25-2.21) *** | 2.65 (2.02-3.48) *** |  |  |
| MI |  |  |  |  |  |  |  |  |
| Yes | 324 | 607 | Ref | 1.32 (0.92-1.87) | 1.75 (1.25-2.44) ** | 2.08 (1.48-2.93) *** | 0. 572 |  |
| No | 376 | 759 | Ref | 1.49 (1.11-2.00) ** | 1.81 (1.34-2.45) *** | 2.71 (2.04-3.60) *** |  |  |
| Angina |  |  |  |  |  |  |  |  |
| Yes | 196 | 355 | Ref | 1.51 (0.96-2.38) | 1.89 (1.21-2.96) ** | 2.88 (1.85-4.48) *** | 0. 889 |  |
| No | 504 | 1011 | Ref | 1.40 (1.08-1.83) * | 1.81 (1.40-2.35) *** | 2.30 (1.79-2.95) *** |  |  |
| DM |  |  |  |  |  |  |  |  |
| Yes | 315 | 585 | Ref | 1.62 (1.15-2.27) ** | 1.71 (1.24-2.37) ** | 2.17 (1.55-3.03) *** | 0. 252 |  |
| No | 385 | 781 | Ref | 1.31 (0.96-1.77) | 1.84 (1.35-2.49) *** | 2.63 (1.97-3.50) *** |  |  |
| TC |  |  |  |  |  |  |  |  |
| >200mg/dl | 213 | 405 | Ref | 1.17 (0.79-1.73) | 1.50 (1.02-2.21) * | 1.87 (1.28-2.73) ** | 0. 409 |  |
| ≤200mg/dl | 487 | 961 | Ref | 1.58 (1.19-2.08) ** | 2.01 (1.53-2.64) *** | 2.76 (2.11-3.60) *** |  |  |
| TG |  |  |  |  |  |  |  |  |
| >150mg/dl | 291 | 592 | Ref | 1.22 (0.86-1.73) | 1.28 (0.90-1.81) | 1.93 (1.38-2.70) *** | 0.036 |  |
| ≤150mg/dl | 409 | 774 | Ref | 1.59 (1.18-2.15) ** | 2.39 (1.79-3.19) *** | 2.90 (2.18-3.87) *** |  |  |

P-value: * <0.05, ** <0.01, *** <0.001

Abbreviations: ABSI, a body shape index; HF, heart failure; CHD, coronary heart disease; MI, myocardial infarction; DM, diabetes mellitus.TC, total cholesterol; TG, triglyceride.

^a^ The other marital includes divorced, widowed, single, and others.

**Figure 1: Flowchart for study population selection.**


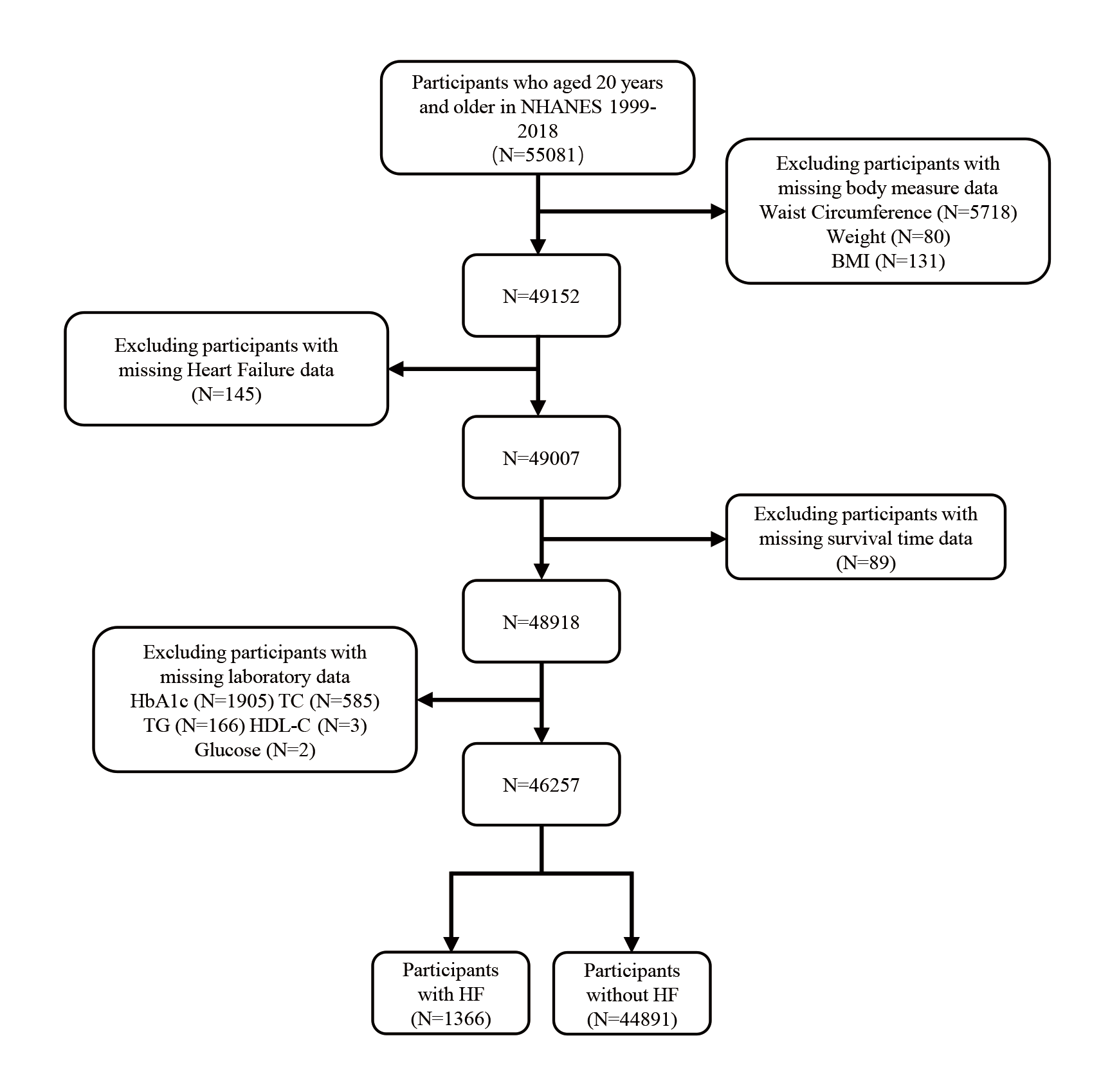


Abbreviation: NHANES, the National Health and Nutrition Examination Survey; BMI, body mass index; HbA1c, hemoglobin A1c; TC, total cholesterol; TG, triglyceride; HDL-C, high-density lipoprotein cholesterol; HF, heart failure.

**Figure 2: K-M survival analysis for all-cause mortality in HF patients.**


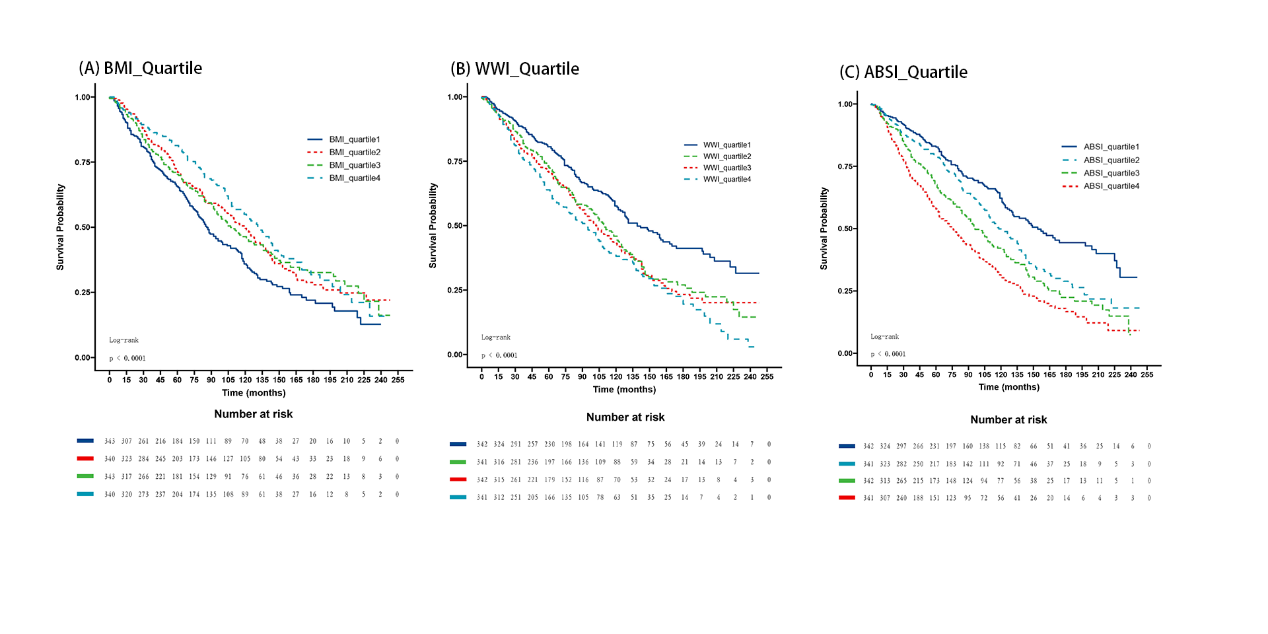


Abbreviations: K-M, Kaplan-Meier; HF, heart failure; BMI, body mass index; WWI, weight-adjusted-waist index; ABSI, a body shape index.

**Figure 3: K-M survival analysis for all-cause mortality in HF patients. (Surrogate adiposity markers without significant differences)**


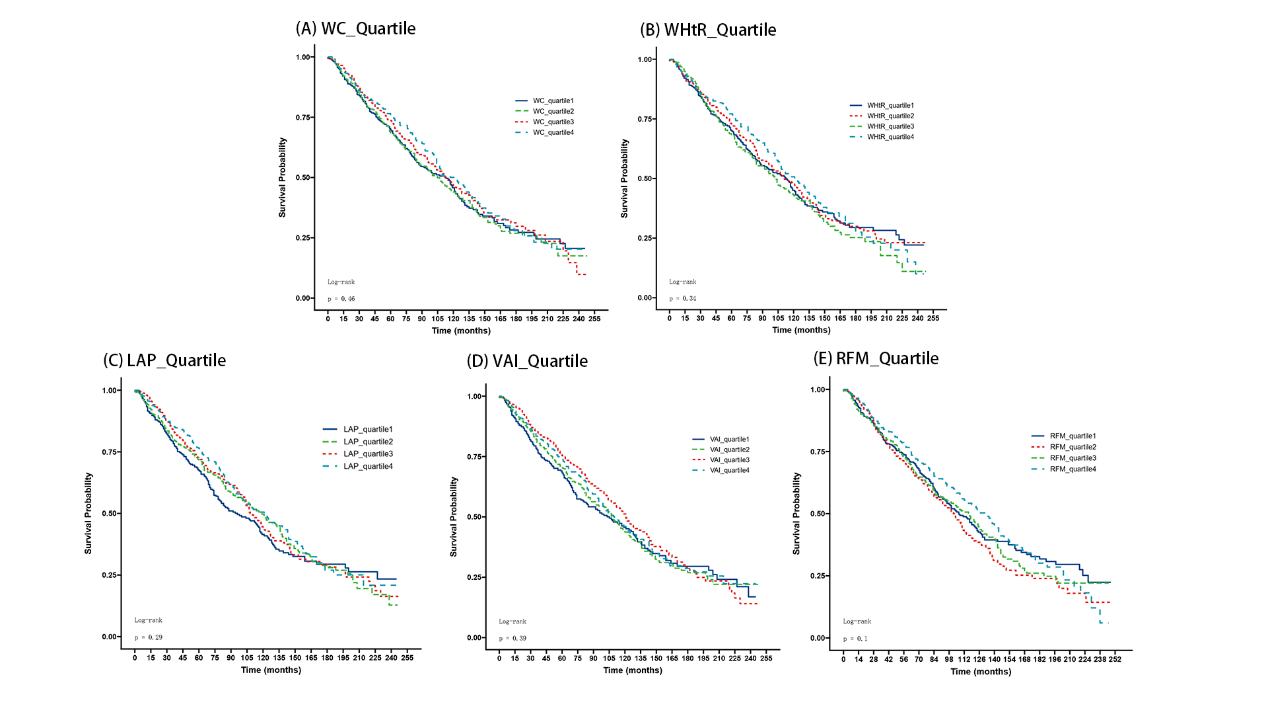


Abbreviations: K-M, Kaplan-Meier; HF, heart failure; WC, waist circumference; WHtR, waist-to-height ratio; LAP, lipid accumulation product; VAI, visceral fat index; RFM, relative fat mass.
